# Job and life satisfaction and preference of future practice locations of physicians on remote islands in Japan

**DOI:** 10.1186/s12960-015-0029-z

**Published:** 2015-05-26

**Authors:** Yoshiaki Nojima, Shunichi Kumakura, Keiichi Onoda, Tsuyoshi Hamano, Kiyoshi Kimura

**Affiliations:** Department of Medical Education and Research, Faculty of Medicine, Shimane University, Izumo, 693-8501 Japan; Oki Hospital, Oki, Japan; Third Division of Internal Medicine, Shimane University, Izumo, Japan; The Center of Health Research and Education, Shimane University, Izumo, Japan; Department of Health and Welfare, Shimane Prefectural Government, Matsue, Japan

**Keywords:** Physician’s satisfaction, Rural and remote islands, Oki islands, Choices of practice location

## Abstract

**Background:**

The objective of this research is to investigate job and life satisfaction and preference of future practice locations of physicians in rural and remote islands in Japan.

**Methods:**

A cross-sectional study was conducted for physicians who reside or resided on the Oki islands: isolated islands situated in the Sea of Japan between the Eurasian continent and the mainland of Japan. A questionnaire was sent to physicians on the Oki islands to evaluate physician satisfaction regarding job environment, career development, living conditions, salary, and support by local government.

**Results:**

Data was analysed for 49 physicians; 47 were male and 2 were female, and the mean ± SD age was 44.3 ± 10.9 years. Among the variables related to physicians’ satisfaction, most of the physicians (>90%) were satisfied with “team work” and “salary”. On the other hand, the majority of physicians (approximately 70%) were not satisfied with the “opportunity to continue professional development”. Age ≥50 years, graduates of medical schools other than Jichi Medical University (established in 1972 with the aim to produce rural physicians), self-selected the Oki islands as a practice location, and satisfaction in “work as a doctor”, “opportunity to consult with peers about patients”, “relationship with people in the community”, and “acceptance by community” were found to be significant factors influencing the choice of the Oki islands as a future practice location. Factors influencing future practice locations on the remote islands were included in a self-reported questionnaire which illustrated the importance of factors that impact both the spouses and children of physicians.

**Conclusions:**

Improving work satisfaction, providing outreach support programmes for career development and professional support in rural practice, and building appropriate relationships between physicians and people in the community, which can in turn improve work satisfaction, may contribute to physicians’ choices of practising medicine on rural and remote islands in Japan. Addressing family issues is also crucial in encouraging the choice of a rural medical practice location.

## Background

A shortage of physicians in rural and underserved areas is an issue of social and political concern in most countries. Geographic maldistribution of physicians is also a critical issue within countries [1]. These issues urgently need to be resolved in order to provide better health-care systems and health outcomes in rural and underserved areas. Japan is facing a shortage of physicians in rural and underserved areas, as well as a maldistribution of physicians between urban and rural areas. The ratio of physicians per 1 000 population in 2010 was estimated as 2.21, which is low among the Organization for Economic Cooperation and Development (OECD) countries (mean ratio, 3.16) [2]. The Japanese government (the Ministry of Education, Culture, Sports, Science and Technology) decided to increase the medical student enrolment quota from 2008, and their numbers increased from 7 625 (in 2007) to 9 041 (in 2013) [3]. However, “OECD Factbook 2014” described that the ratio of new medical graduates per 100 000 population in Japan in 2011 (or latest available year) was 5.97 with a ranking second to the lowest among OECD countries (mean ratio, 10.56) [2]. It has also been reported that the estimated supply of physicians in Japan will not be sufficient for the demand for health care in the ageing society [4], because the Japanese population is ageing rapidly, and consequently, the demand for health care is increasing. Moreover, the shortage of rural physicians in rural and underserved areas and the subsequent maldistribution of physicians accelerated after the introduction of the new mandatory 2-year postgraduate training system in 2004. The postgraduate training system is aimed at improving clinical competencies of residents in primary care and requires medical school graduates to choose their own training location through the matching service between graduates and clinical training facilities. Most clinical training facilities, such as academic hospitals, are located in urban areas, rather than in rural areas, and as a result, the graduates tend to choose their location in an urban area. In addition, it became evident that the number of physicians working at hospitals has been significantly increasing in urban areas but has not been increasing in areas with low-population densities in Japan [5]. The newly introduced postgraduate training system therefore accelerated physician shortages in rural areas and expanded the maldistribution of physicians.

On the other hand, the Jichi Medical University (JMU) was established in Japan in 1972 with the explicit aim to produce rural physicians. JMU admission policy is closely linked to the students’ home prefecture. Japan has 47 prefectures, and JMU enrols two or three students from each prefecture every year. Overall, approximately 100 students are admitted to JMU per year, and all of the students receive a scholarship, which is fully funded by their home prefectural government. The graduates are required to provide 9 years of work in their home prefecture, including a 5 to 6 years obligation in rural dispatch areas. It has been reported that 69.8% of JMU graduates remained in their home prefectures for at least 6 years after their obligatory service [6]. Since 2008, when the government decided to increase the medical student enrolment quota, many medical schools in Japan have begun a special admission programme with the aim to supply rural physicians. Medical schools enrol students highly motivated for future rural work. A majority of those students are provided a scholarship funded by their prefectural government. As of 2013, this rural admission programme was introduced in 68 of 79 medical schools, in addition to JMU, and 1 425 students were enrolled (including students with a scholarship related to future rural work in their home prefecture) [7]. The efficacy of this admission programme in improving the shortage of physicians in rural and underserved areas in Japan is currently under investigation.

There have been several reports dealing with evaluations by rural physicians and evaluations on the professional isolation of island physicians, in addition to a comparison between island and mainland rural practices in Japan [8–13]. Inoue et al. conducted a comparative study of rural clinics in remote Japanese islands and mainland areas and showed that physicians in remote island clinics have less medical training than mainland physicians [8]. More than half of the clinic physicians in remote islands have no regular training schedule, in contrast to less than a quarter of the inland clinic physicians. Rural physicians were reported to be less satisfied with the distance to major cities, the municipal government’s attitude, and *locum* availability [9]. It has also been described that the attitude of the people in the community and the satisfaction of family members were important factors for rural physicians [10]. On the other hand, a survey of a total of 4 896 doctors working for 828 public clinics and hospitals in Japan showed that postgraduate training in general internal medicine, general surgery, anesthesiology, paediatrics, and gastroenterology were positively related with the intention to continue a rural career [11]. Matsumoto et al. analysed the characteristics of JMU graduates and showed that having a rural upbringing and the choice of primary care specialties were positively associated with graduates having a rural address and settlement [12]. Despite these studies, job and life satisfaction and preference of future practice locations of physicians in rural and remote islands in Japan are not well understood.

**Fig. 1:**
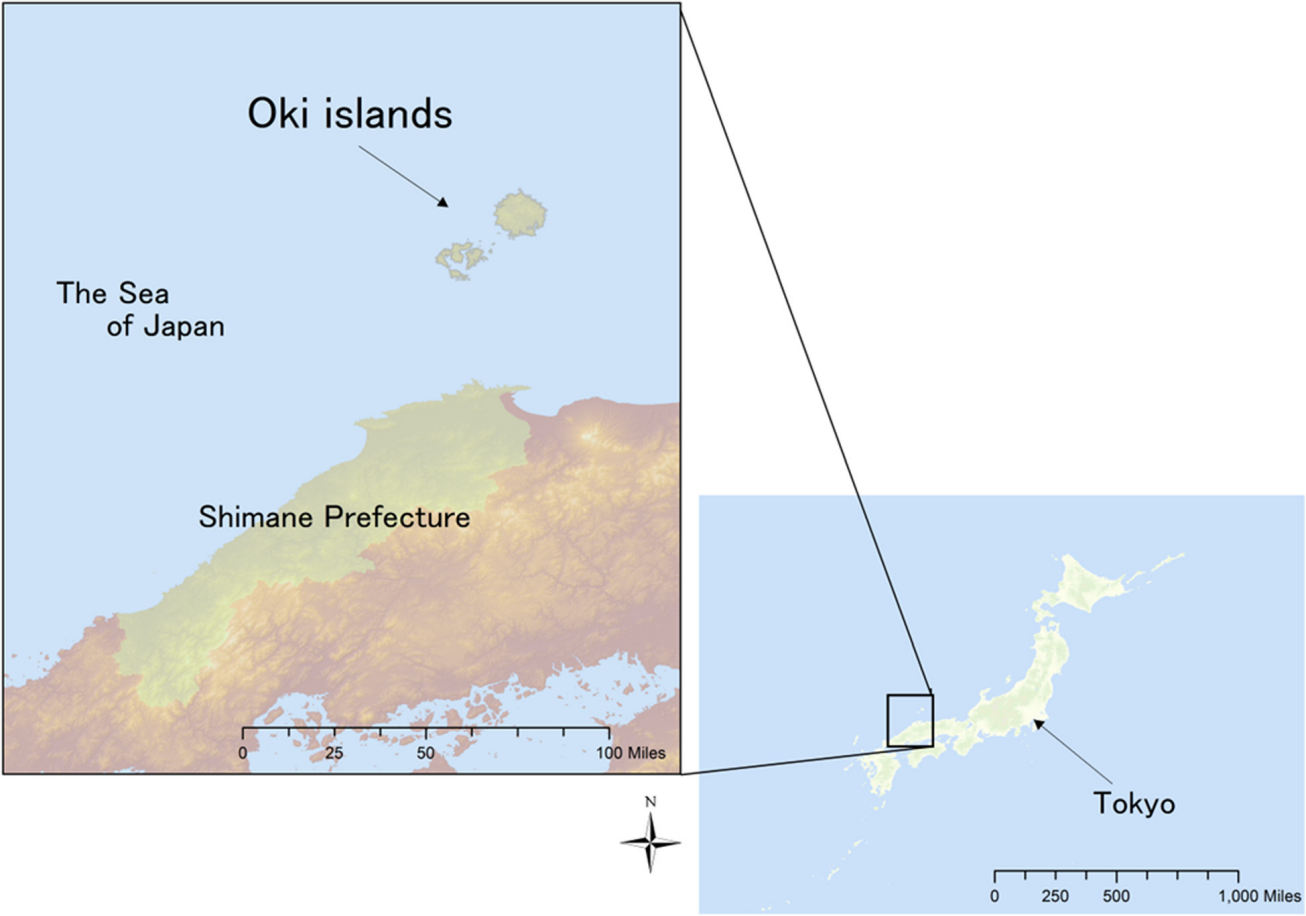
Map of the Oki islands. The Oki islands are rural and remote islands situated in the Sea of Japan between the Eurasian continent and mainland of Japan. The Oki islands belong to the Shimane Prefecture, Japan

The Oki islands are rural and isolated islands situated in the Sea of Japan between the Eurasian continent and the mainland of Japan (Fig. 1). The population of the islands is approximately 15 000 people. The ratio of physicians per 1 000 populations is low on the Oki islands as compared with the mean ratio in Japan (1.57 versus 2.38) [14]. In this study, we examined job and life satisfaction and preference of future practice locations of physicians working on the Oki islands by using a self-administered questionnaire.

## Methods

### Study population

The data investigated in this study were drawn from a cross-sectional study of physicians on the Oki islands in Japan. The study was conducted in the Department of Medical Education and Research, Faculty of Medicine, Shimane University, in cooperation with the Department of Health and Welfare, Shimane prefectural government. We enrolled physicians who resided on the Oki islands in 2012 and/or resided before 2012. Physicians were considered eligible if they were working or have worked full time. It was not relevant whether they were working or have worked in a hospital or in a clinic as specialists or as general physicians (general practitioner).

### Evaluation of physicians’ satisfactions and factors influencing future practice location

Physicians’ satisfaction related to their job and life on remote and rural islands was evaluated by using a self-administered questionnaire. The questionnaire variables included demographic background (age, sex, upbringing, medical school attended, and reasons for choosing their medical practice and their medical practice career on the Oki islands), job and work environment on the Oki islands (work as a doctor, relationship with patients, relationship with people in the community, opportunity to consult with peers about patients, working hours/condition, recruitment of *locum tenens*, team work, medical clerks, cooperation with other medical facilities, teleradiology services, air medical services, and medical instruments), career development (attendance at academic meetings and opportunities to continue professional development), living conditions (housing conditions, schooling for children, leisure, procurement of daily necessities, acceptance by community, and support in daily life), salary, and coordination and support by local government. Each variable, except for demographic background, was responded to by using a four-point Likert scale (“satisfactory”, “fairly satisfactory”, “not so satisfactory”, and “not at all satisfactory”). The questionnaire was addressed to the physicians regardless of whether they preferred the Oki islands as a future practice place or not. Subsequently, variables were compared between physicians who preferred the Oki islands as a future practice location and physicians who did not to determine factors influencing the choice of a future practice location.

The questionnaire provided additional variables that quantify factors influencing the choice of practice location (spouse’s needs, parent’s needs, schooling for children, self-professional development, obligation tied to scholarships or rural or urban dispatches from a medical university) with the use of a four-point Likert scale (“influenced”, “fairly influenced”, “not so influenced”, and “not at all influenced”). The degree of influence of variables on the choices of practice location was also compared between physicians who preferred the Oki islands as a future practice place and those who did not in order to identify factors which were closely associated with the choice of practising medicine on rural and remote islands.

Data collection took place during September and October 2012. Participants gave written informed consent, and data were collected with permission stipulating use for research purposes only.

### Data analysis

Data were analysed according to the Likert scale. The descriptive aspect of analysis measured the frequencies by which the respondents gave a rating of “satisfactory” or “fairly satisfactory” and “influenced” or “fairly influenced” to a specific question and the corresponding mean score. The scores were calculated as “satisfactory” = 3, “fairly satisfactory” = 2, “not so satisfactory” = 1, and “not at all satisfactory” = 0 or as “influenced” = 3, “fairly influenced” = 2, “not so influenced” = 1, and “not at all influenced” = 0.

### Statistical analysis

We assessed differences in baseline demographic characteristics and variables according to the study group with the use of chi-square test. A *p* value of <0.05 was considered to indicate statistical significance.

## Results

### Demographic characteristics

Seventy-five physicians who worked between 1980 and 2012 on the Oki islands were identified, and 49 (65.3%) responded to the questionnaire. In the survey, physicians who worked from 1980 onward were included, since physicians’ data before 1980 were not available.

The characteristics of physicians were shown in Table 1. The mean age ± SD was 44.3 ± 10.9 years. Forty-seven physicians were male, and two were female. Fifteen physicians (30.6%) and 7 spouses (14.2%) had rural backgrounds. Thirty-six physicians (73.5%) graduated from JMU, and of the remaining 13, 5 had graduated from Shimane University and 8 had graduated from other universities.

**Table 1:** Characteristics of physician (*n* = 49)

	*n* (%)
Age (years)	
mean ± SD	44.3 ± 10.9
<30	4 (8.2)
30–39	19 (38.8)
40–49	9 (18.4)
50–59	10 (20.4)
≥60	7 (14.3)
Sex	
Male	47 (95.9)
Female	2 (4.1)
Upbringing	
Rural	15 (30.6)
Urban	34 (69.4)
Spouse’s upbringing	
Rural	7 (14.2)
Urban	41(83.7)
Medical school attended	
Jichi Medical University	36 (73.5)
Other universities^a^	13 (26.5)
Reason for choosing practice place on the Oki islands	
Obligation after graduation^b^	38 (77.6)
Self-willing	11 (22.4)
Practice career on the Oki islands (years)	
<1	8 (16.3)
1–4	24 (49)
5–10	12 (24.5)
10–19	15 (10.2)

^a^Other universities than Jichi Medical University

^b^Corresponding to a period of mandatory work depending upon the receipt of scholarship (*n* = 36)

Thirty-six physicians (73.5%) chose the Oki islands as a place to practice medicine due to an obligation after graduation, depending upon the receipt of scholarships. Two (4.1%), who did not receive scholarships, chose the Oki islands by responding to a request of the hospital located in the islands. Eleven (22.4%) had chosen Oki islands as a place to practice medicine due to their own willingness to practice there. The mean length of medical practice of physicians on the Oki islands was 4.9 years (mean ± SD, 4.9 ± 4.5).

### Physicians’ satisfactions in remote and rural islands

The degree of satisfaction was evaluated by analysing the scores for each variable (Table 2). Among the variables, “team work” and “salary” were found to have the highest satisfaction score. Ninety-four percentage and 91.1% of physicians were satisfied with “team work” and “salary”, respectively. A majority of physicians were also satisfied with “air medical service”, “acceptance by the community”, “work as a doctor”, and “relationship with patients”. On the other hand, the degree of satisfaction for “opportunity to continue professional development” was shown to be the lowest. The second lowest was “providing medical clerks”. The percentages of physicians who were satisfied with “opportunity to continue professional development” and “providing medical clerk” were 32.6% and 41.5%, respectively.

**Table 2:** The degree of physician’s satisfactions on the Oki islands

	Physician who are satisfied^a^							
			Grown up place			Medical university		
	Total		Rural	Urban	*p* value^b^	JMU^c^	Other than JMU	*p* value^d^
	*n*/total *n* (%)	Score^e^ (mean ± SD)	*n*/total *n* (%)	*n*/total *n* (%)		*n*/total *n* (%)	*n*/total *n* (%)	
Job and work environment								
Professional identity as a doctor	43/49 (87.8)	2.2 ± 0.8	13/15 (86.7)	30/34 (88.2)	ns	31/36 (86.1)	12/13 (92.3)	ns
Relationship with patients	39/46 (84.8)	2.2 ± 0.8	13/15 (86.7)	26/31 (83.9)	ns	28/35 (80.0)	11/11 (100.0)	ns
Relationship with people in the community	36/45 (80.0)	2.1 ± 0.8	11/14 (78.6)	25/31 (80.6)	ns	27/35 (77.1)	9/10 (90.0)	ns
Opportunity to consult with peers about patients	32/46 (69.6)	1.9 ± 0.9	12/15 (80.0)	20/31 (64.5)	ns	25/35 (71.4)	7/11 (63.6)	ns
Working hours/condition	28/46 (60.9)	1.7 ± 0.9	12/15 (80.0)	16/31 (51.6)	ns	21/35 (60.0)	7/11 (63.6)	ns
Recruitment of *locum tenens*	25/47 (53.2)	1.5 ± 1	8/15 (53.3)	17/32 (53.1)	ns	19/35 (54.3)	6/12 (50.0)	ns
Team work	43/46 (93.5)	2.4 ± 0.6	15/15 (100.0)	28/31 (90.3)	ns	33/35 (94.3)	10/11 (90.9)	ns
Medical clerks	17/41 (41.5)	1.3 ± 1	6/14 (42.9)	11/27 (40.7)	ns	11/30 (36.7)	6/11 (54.5)	ns
Cooperation with other medical facilities	37/46 (80.4)	2.1 ± 0.8	14/15 (93.3)	23/31 (74.2)	ns	27/35 (77.1)	10/11 (90.9)	ns
Teleradiology service	29/45 (64.4)	1.7 ± 0.9	9/15 (60.0)	20/30 (66.7)	ns	24/34 (70.6)	5/11 (45.5)	ns
Air medical service	41/46 (89.1)	2.3 ± 0.8	13/15 (86.7)	28/31 (90.3)	ns	32/35 (91.4)	9/11 (81.8)	ns
Medical instruments	29/46 (63.0)	1.7 ± 0.8	11/15 (73.3)	18/31 (58.1)	ns	22/35 (62.9)	7/11 (63.6)	ns
Career development								
Attendance to academic meetings	23/45 (51.1)	1.4 ± 0.9	6/14 (42.9)	17/31 (54.8)	ns	19/35 (54.3)	4/10 (40.0)	ns
Opportunity to continue professional development	15/46 (32.6)	1.2 ± 0.9	3/15 (20.0)	12/31 (38.7)	ns	13/35 (37.1)	2/11 (18.2)	ns
Living conditions								
Housing conditions	36/46 (78.3)	2 ± 0.9	11/15 (73.3)	25/31 (80.6)	ns	26/35 (74.3)	10/11 (90.9)	ns
Schooling for children	23/40 (57.5)	1.7 ± 0.9	5/12 (41.7)	18/28 (64.3)	ns	17/30 (56.7)	6/10 (60.0)	ns
Leisure	29/46 (63.0)	1.7 ± 0.8	8/15 (53.3)	21/31 (67.7)	ns	24/35 (68.6)	5/11 (45.5)	ns
Procurement of daily necessities	22/46 (47.8)	1.5 ± 0.8	4/15 (26.7)	18/31 (58.1)	ns	16/35 (45.7)	6/11 (54.5)	ns
Acceptance by community	39/45 (86.7)	2.3 ± 0.8	14/15 (93.3)	25/30 (83.3)	ns	28/34 (82.4)	11/11 (100.0)	ns
Support in daily life	32/45 (71.1)	1.9 ± 0.9	13/15 (86.7)	19/30 (63.3)	ns	23/34 (67.6)	9/11 (81.8)	ns
Salary	41/45 (91.1)	2.4 ± 0.7	12/14 (85.7)	29/31 (93.5)	ns	32/34 (94.1)	9/11 (81.8)	ns
Coordination and support by local government	31/46 (67.4)	1.8 ± 0.7	10/15 (66.7)	21/31 (67.7)	ns	23/35 (65.7)	8/11 (72.7)	ns

*ns* not significant

^a^Answered “satisfactory” or “fairly satisfactory”. Data not available on all subjects

^b^Rural versus urban

^c^JMU = Jichi Medical University

^d^JML versus other than JMU

^e^Calculate as “satisfactory” = 3, “fairly satisfactory” = 2, “not so satisfactory” = 1, “not at all satisfactory” = 0

We then compared the degree of satisfaction for each variable between physicians with a rural upbringing versus an urban upbringing and found no significant difference between the two groups (Table 2). Moreover, no significant difference in the degree of satisfaction was found between physicians who graduated from JMU versus other universities (Table 2).

### Factors influencing choices of practice locations

Our survey yielded 25 physicians who favourably indicated the Oki islands as a future place to practice medicine, while 18 did not. We investigated the relationship between the subjects’ background and the likeliness of the Oki islands being a future place to practice medicine. As shown in Table 3, the number of physicians who favoured the Oki islands as a future place to practice medicine was significantly greater in populations aged ≥50 years than in those aged <50 years (90.9% versus 46.9%; *p* < 0.05). Forty-five percent of physicians who graduated from JMU answered favourably for the Oki islands as a future place to practice medicine, as compared with 91.7% of those who graduated from medical schools other than JMU (*p* < 0.01). The percentage of physicians favouring the Oki islands as a future place to practice medicine was significantly greater in populations who self-willingly chose the Oki islands, as compared with those who did so due to an obligation (100% versus 45.5%, *p* < 0.01). In addition, our survey data indicated that the physicians who self-willingly choose the Oki islands were graduates of medical schools other than JMU.

**Table 3:** Factors favouring the Oki islands as future practice location

	Favour the Oki islands as future practice location^a^			
	Yes (*n* = 25)	No (*n* = 18)	Rate^b^	*p* value
	*n*	*n*	%	
Age (years)				
<40	11	11	50.0	ns
≥40	14	7	66.7	
<50	15	17	46.9	<0.05
≥50	10	1	90.9	
Sex				
Male	25	16	61.0	ns
Female	0	2	0.0	
Upbringing				
Rural	7	6	53.8	ns
Urban	18	12	60.0	
Oki islands	4	0	100.0	ns
Other than Oki islands	21	18	53.8	
Spouse’s upbringing				
Rural	2	5	28.6	ns
Urban	23	13	63.9	
Medical school attended				
JMU^c^	14	17	45.2	<0.01
Other than JMU	11	1	91.7	
Reason for choosing practice place on Oki islands				
Obligation	15	18	45.5	<0.01
Self-willing	10	0	100.0	
Practice career on Oki islands (years)				
<5	13	12	52.0	ns
≥5	12	6	66.7	
Professional identity as a doctor				
Satisfied^d^	24	13	64.9	<0.05
Not satisfied^e^	1	5	16.7	
Relationship with patients				
Satisfied^d^	21	13	61.8	ns
Not satisfied^e^	1	5	16.7	
Relationship with people in the community				
Satisfied^d^	20	11	64.5	<0.05
Not satisfied^e^	2	17	22.2	
Opportunity to consult with peers about patients				
Satisfied^d^	18	9	66.7	<0.05
Not satisfied^e^	4	9	30.8	
Frequency of duty				
Satisfied^d^	16	10	61.5	ns
Not satisfied^e^	8	8	50.0	
Recruitment of *locum tenens*				
Satisfied^d^	12	9	57.1	ns
Not satisfied^e^	12	8	60.0	
Team work				
Satisfied^d^	21	16	56.8	ns
Not satisfied^e^	1	2	33.3	
Medical clerks				
Satisfied^d^	11	4	73.3	ns
Not satisfied^e^	10	10	50.0	
Cooperation with other medical facilities				
Satisfied^d^	19	13	59.4	ns
Not satisfied^e^	3	5	37.5	
Teleradiology service				
Satisfied^d^	16	9	64.0	ns
Not satisfied^e^	6	8	42.9	
Air medical service				
Satisfied^d^	20	15	57.1	ns
Not satisfied^e^	2	3	40.0	
Medical instruments				
Satisfied^d^	16	10	61.5	ns
Not satisfied^e^	6	8	42.9	
Attendance to academic meetings				
Satisfied^d^	11	8	57.9	ns
Not satisfied^e^	11	10	52.4	
Opportunity to continue professional development				
Satisfied^d^	7	5	58.3	ns
Not satisfied^e^	15	13	53.6	
Housing conditions				
Satisfied^d^	17	14	54.8	ns
Not satisfied^e^	5	4	55.6	
Schooling for children				
Satisfied^d^	15	7	68.2	ns
Not satisfied^e^	7	8	46.7	
Leisure				
Satisfied^d^	16	9	64.0	ns
Not satisfied^e^	6	9	40.0	
Procurement of daily necessities				
Satisfied^d^	12	6	66.7	ns
Not satisfied^e^	10	12	45.5	
Acceptance by community				
Satisfied^d^	21	12	63.6	<0.05
Not satisfied^e^	1	5	16.7	
Support in daily life				
Satisfied^d^	16	10	61.5	ns
Not satisfied^e^	6	7	46.2	
Salary				
Satisfied^d^	20	15	57.1	ns
Not satisfied^e^	1	3	25.0	
Coordination and support by local government				
Satisfied^d^	14	13	51.9	ns
Not satisfied^e^	8	5	61.5	

*ns* not significant

^a^Data not available on all subjects

^b^The ratio of number of physicians who prefer the Oki islands as future practice location to total number

^c^JMU = Jichi Medical University

^d^Answered “satisfactory” or “fairly satisfactory”

^e^Answered “not so satisfactory” or “not at all satisfactory”

Next, we examined the relationship between physician’s satisfaction and likeliness of the Oki islands as a future place to practice medicine. The number of physicians favouring the Oki islands was found to be significantly greater in the population who were satisfied with “work as a doctor”, “opportunity to consult with peers about patients”, “relationship with community people”, and “acceptance by community”, as compared with the population who were not satisfied with these variables (64.9% versus 16.7% [*p* < 0.05], 64.5% versus 22.2% [*p* < 0.05], 66.7% versus 30.8% [*p* < 0.05], and 63.6% versus 16.7% [*p* < 0.05], respectively). This result suggests that satisfaction in “work as a doctor”, “opportunity to consult with peers about patients”, “relationship with community people”, and “acceptance by community” were significant factors influencing choices of practice locations.

### Self-reported factors influencing future practice location

We asked physicians to provide variables that influenced their choice of future place to practice medicine. As shown in Table 4, 93.5% of physicians reported to be influenced by “spouse’s needs”, 91.3% by “schooling for children”, and 80.4% by “parent’s needs”. Approximately half of the physicians (56.3%) interviewed were found to be influenced by “whether the medical practice was urban or rural”. Then, we compared these variables between physicians who preferred the Oki islands as a future practice place and physicians who did not (Table 5). The number of physicians who were influenced or not influenced by “spouse’s needs” did not differ significantly between the two groups. Similarly, no significant difference between the number of physicians who were influenced or not influenced by “schooling for children”, “parent’s needs”, “obligation/dispatch”, “opportunity to professional development”, or “whether the medical practice was urban or rural” was found between the two groups.

**Table 4:** Factors influencing the choice of practice location^a^

	*n* ^b^/total *n* (%)	Score^c^ (mean ± SD)
Spouse’s needs	43/46 (93.5)	2.5 ± 0.7
Schooling for children	42/46 (91.3)	2.4 ± 0.8
Parent’s needs	37/46 (80.4)	2 ± 0.8
Obligation after graduation^d^	29/45 (64.4)	1.7 ± 1
Opportunity to professional development	28/48 (58.3)	1.7 ± 1
Rural/urban	27/48 (56.3)	1.6 ± 1

^a^Data not available on all subjects

^b^The number of physicians who answered to be “influenced” or “fairly influenced” in choosing the Oki islands as their practice place

^c^Calculated as “influenced” = 3, “fairly influenced” = 2, “not so influenced” = 1, and “not at all influenced” = 0

^d^Mandatory work upon the receipt of scholarships

**Table 5:** Factors influencing the choice of practice location: comparison between physicians who prefer the Oki islands as future practice location and those who do not^a^

	Favour the Oki islands as future practice location			
	Yes (*n* = 25)	No (*n* = 18)	Rate^b^	*p* value
	*n*	*n*	%	
Spouse’s needs				
Influenced^c^	22	16	57.9	ns
Not influenced^d^	0	2	0.0	
Schooling for children				
Influenced^c^	21	16	56.8	ns
Not influenced^d^	1	2	33.3	
Parent’s needs				
Influenced^c^	19	14	57.6	ns
Not influenced^d^	3	6	33.3	
Obligation after graduation^e^				
Influenced^c^	14	11	56.0	ns
Not influenced^d^	7	7	50.0	
Opportunity to professional development				
Influenced^c^	13	11	54.2	ns
Not influenced^d^	12	6	66.7	
Rural/urban				
Influenced^c^	13	11	54.2	ns
Not influenced^d^	12	6	66.7	

*ns* not significant

^a^Data not available on all subjects

^b^The ratio of number of physicians who prefer the Oki islands as future practice location to total number

^c^Answered “influenced” or “fairly influenced”

^d^Answered “not so influenced” or “not at all influenced”

^e^Mandatory work upon the receipt of scholarships

## Discussion

In this study, we evaluated physicians’ satisfaction related to work and life on the remote and rural Oki islands through a self-administered questionnaire. Among the variables, “team work” and “salary” showed the highest satisfaction scores, and other variables having high-satisfaction scores included “air medical service”, “acceptance by community”, “work as a doctor”, and “relationship with patients”. Teamwork is one of the crucial elements for medical practice in remote and underserved areas, which is often lacking for an absolute number of health-care workers. The fact that most physicians (>90%) were satisfied with “team work” suggests that a rural medical practice on the Oki islands provides an excellent teamwork environment for health-care workers. Moreover, the Oki islands might offer opportunities for a very good close relationship between physicians and the community, since a majority of physicians were highly satisfied with “acceptance by community” and “relationship with patients”. The excellence in teamwork and relationships with the community is characteristic to rural practice on the Oki islands. We believe this point should be appealed to when promoting the recruitment of physicians on the Oki islands.

On the other hand, the majority of physicians (approximately 70%) were not satisfied with “opportunity to continue professional development”, and this satisfaction score remained the lowest among the variables. Similar to our results, White et al. described the importance of continuing professional education for rural and remote doctors in Queensland [15]. In this study, 94% of 429 doctors answered “agree” or “strongly agree” that access to continuing professional education contributes to confidence in practising in rural and/or remote locations. In addition, data showed that 80% “agree” or “strongly agree” that they were less likely to remain without access to continuing professional education. To provide an adequate opportunity to continue professional development is crucial for rural physicians because continuing professional development increases professional satisfaction, competencies, and efficiency.

It is evident that physicians with a rural background are twice as likely to work in rural practice when compared to physicians with an urban background [16]. Based on this fact, we compared physicians’ satisfaction between a rural upbringing versus an urban upbringing (Table 2). There was no significant difference in the percentage of physicians who were satisfied with job and work environment, career development, living conditions, salary, and coordination or support by local government between those with a rural upbringing and those with an urban upbringing. Similarly, physicians’ satisfaction as measured by these variables was found not to differ significantly between JMU graduates and non-JMU graduates. The physicians’ satisfaction in rural practice was not, at least in part, influenced by their backgrounds, such as their upbringing or medical school attended.

Our survey presented here showed that non-JMU graduates were more likely to choose the Oki islands than those who graduated from JMU (91.7% versus 45.2%; *p* < 0.01). This result was closely associated with the self-selected location of medical practice. Among the physicians who self-willingly chose the Oki islands for their medical practice location, all of them favoured the Oki islands for their future, which indicated that a self-selection of practice location is a strong predictor of future practice location. There was a very high tendency in the choice of the Oki islands as future location observable in non-JMU graduates. In contrast, approximately half of the physicians (45.2%) who graduated from JMU favoured the Oki islands as a future practice location. It remained unclear whether this rate is high for physicians with compulsory service obligations, which warrants further research; however, we should continue to strive for more satisfactory results.

In our present study, we addressed factors associated with physicians’ choices of practice locations in remote and rural islands in Japan and found that satisfaction of “work as a doctor”, “opportunity to consult with peers about patients”, “relationship with community people”, and “acceptance by community” were positively correlated with likeliness of choosing the Oki islands as a future location to practice medicine (Table 3). Job satisfaction has been shown to be associated with work commitment among health workers [17, 18]. Job satisfaction may thus be a motivating factor for strengthening professional identity as a rural physician.

Moreover, we found that rural physicians in the Oki islands were not satisfied with “opportunity to consult with peers about patients”. Dieleman et al. examined health workers’ job perception and motivation in Vietnam and identified “appreciation and support by managers and colleagues” as a factor for motivation [18]. Establishing a support system among physicians in rural practice is therefore effective in encouraging a physician’s motivation, as well as reducing feelings of professional isolation in rural settings.

In this study, we also found that “relationship with people in the community” and “acceptance by the community” are significant factors influencing the future choice of a medical practice location. Better relationships with the community may enhance job satisfaction of physicians in daily practice, since better relationships between physicians and patients in the community are a fundamental element for a practising physician. Thus, building better relationships between physicians and the community is an effective strategy to promote rural commitment of physicians on both remote and rural islands. Rural physicians usually take care of not only treating diseases but also lifestyle, family, housing, and other circumstances surrounding the patient. Therefore, physicians who practice in rural and underserved communities can usually develop strengthened and better physician–patient relationships. Oki islands may be characteristic in providing better relationships between physicians and the community, which can be an effective appeal for physicians to work in isolated islands.

On the other hand, many physicians mentioned family issues as important factors influencing choices of practice location. “Spouse’s needs” was most frequently mentioned to influence choice of practice location, followed by “schooling for children” (Table 4). A comparison of the variables, including “spouse’s needs” and “schooling for children”, between physicians who preferred the Oki islands as a future practice place and those who did not showed that the variables’ degree of influence on the choice of practice location did not differ between the two groups (Table 5). Therefore, in spite of whether or not physicians preferred the Oki islands as a future practice place, “spouse’s needs” and “schooling for children” could be crucial factors influencing the choice of practice locations for physicians. A majority of spouses grow up in urban areas (83.7%; Table 1), and thus, support for spouses in daily life and other aspects may promote physicians’ choice of rural practice. Moreover, in Japan, children’s education is one of the most serious issues for parents, and our study clarified that more than 90% of physicians reported “schooling for children” as an influencing factor for choosing practice locations. Thus, to provide a better education system for children in rural and underserved areas may contribute to increase motivation to become a rural physician. This issue should be addressed further.

### Limitations

Our present study had some limitations. First, we drew conclusions based on a comparatively small number of participants in this study. For example, no significant differences of physicians’ satisfaction in rural practices were found between physicians with a rural upbringing versus an urban upbringing (Table 2), which may be related to the small sample size. Second, the participants had heterogeneity in their backgrounds, regarding JMU and non-JMU graduates. JMU graduates had an obligation of 9 years rural practice, while non-JMU graduates did not have a rural obligation, and therefore, a majority chose their practice place on their own willingness, creating some differences in favour of choosing their practice place for the future. Finally, we evaluated factors relating to choices of practice location (Table 4); however, no comparisons were made between island and non-island physicians. This is a difference from the previous study conducted by Inoue et al. [8]. In order to remove this limitation, we would like to make a comparison between island and non-island physicians in the future.

## Conclusions

The results of our survey indicate that approaches to improve work satisfaction, and develop career development programmes and professional support, are crucial for encouraging the choices of practice location in remote and rural islands in Japan. Further, to build better relationships between physicians and their community, which may in turn improve work satisfaction, and to address family issues also contribute to improve shortages of rural physicians. These approaches are beneficial to promote the choice of rural practice, but are not exhaustive for resolving physician shortages in rural and underserved areas and the maldistribution of physicians in Japan. Unfortunately, we do not have any definite criteria about an appropriate number of physicians (e.g. general physicians and/or specialists) that is required for the physician-shortage areas, as well as Japan as a whole, and we do not define how to control the distribution of physicians to physician-shortage areas. Moreover, there is no compulsory regulation in the geographic distribution of physicians, and physicians are free to choose their own work location as they see fit. Japan has now changed the admission policies to increase the number of medical students enrolled who are motivated to practice medicine in a rural area in the future. Therefore, a revision and strengthening of both undergraduate and postgraduate programmes so as to increase motivation for rural practice is needed thereby increasing the number of rural physicians. In addition to the approaches identified by our results, building a better system that promotes education, the foundation for producing competent rural physicians, and regulating the geographic distribution of physicians, is required. Our goal is to improve health outcomes in the community through the building of a better physician and health system.
